# Sustainable Designed Pavement Materials

**DOI:** 10.3390/ma13071575

**Published:** 2020-03-29

**Authors:** Yue Xiao, Sandra Erkens, Mingliang Li, Tao Ma, Xueyan Liu

**Affiliations:** 1 Key Laboratory of Transport Industry of Road Structure and Material, Research Institute of Highway (RIOH), Ministry of Transport, Beijing 100088, China; xiaoy@whut.edu.cn; 2 State Key Laboratory of Silicate Materials for Architectures, Wuhan University of Technology, Wuhan 430070, China; 3 Faculty of Civil Engineering and Geosciences, Delft University of Technology, 2628 CN Delft, The Netherlands; s.m.j.g.erkens@tudelft.nl (S.E.); X.Liu@tudelft.nl (X.L.); 4 School of Transportation, Southeast University, Nanjin 210096, China; matao@seu.edu.cn

**Keywords:** pavement materials, sustainable designed pavement materials, recycling, recycled pavement materials, ageing resistance, modified asphalt materials, rejuvenator, skid resistance, pavement surfacing

## Abstract

This Special Issue “Sustainable Designed Pavement Materials” has been proposed and organized as a means to present recent developments in the field of environmentally-friendly designed pavement materials. For this reason, articles included in this special issue relate to different aspects of pavement materials, from industry solid waste recycling to pavement materials recycling, from pavement materials modification to asphalt performance characterization, from pavement defect detection to pavement maintenance, and from asphalt pavement to cement concrete pavement, as highlighted in this editorial.

This Special Issue “Sustainable Designed Pavement Materials” has been proposed and organized as a means to present recent developments in the field of environmentally-friendly designed pavement materials. It covers a wide range of selected topics on pavement materials. In total, 40 papers passed the peer-review and got published in this Special Issue. Universities and institutes considered as the most successful organizations, such as Wuhan University of Technology (10 papers), Southeast University (7 papers), Changsha University of Science & Technology (7 papers), Chang’an University (6 papers), Delft University of Technology (3 papers), Harbin Institute of Technology (2 papers), RWTH Aachen University (2 papers), Pennsylvania State University, Washington State University, Purdue University, and Michigan Technological University, have contributed a lot to this Special Issue. A brief summary of the articles is given in this editorial.

Research on solid waste recycling in pavement materials is considered as one of the most economic ways to achieve sustainable designed pavements. Kong et al. [1,2] studied the possibility of using oxygen furnace slag filler in asphalt mixture, and the BOF (Basic Oxygen Furnace) slag coarse aggregate was also presented in his research for making asphalt concrete. Three types of BOF slag fillers were concluded in their research. Ye et al. [3] investigated the effects of different cooling and treatment processes on the morphological features of BOF steel slag, and the effect of slag morphologies on the performance of asphalt mixtures. Another article from Qian et al. [4] also focused on slag pavement materials. Phosphorous slag was used as asphalt mixture aggregates on cement concrete deck to improve the interface bonding strength. Quarry fines were proposed by Zhang et al. [5,6] for pavement construction materials, by evaluating the properties of basic quarry fines and stabilized quarry fine specimens prepared using the gyratory compactor. Besides using slags as aggregates, crumb rubber was another widely used solid waste in pavement materials. The short-term aging of microwave activation crumb rubber was studied by Li et al. [7], the mixing and compaction temperatures of the crumb rubber modified asphalt mixture were discussed in the research from Li et al. [8], while Gu et al. [9] developed a meso-structure-based finite element model of rubber modified mixture to predict both the dynamic modulus and phase angle properties.

Reuse of pavement materials is another eco-efficient method for sustainable pavement design. Long-term ageing resistance and healing properties of pavement materials were discussed in detail. The ageing characteristics of asphalt materials during their service life were evaluated by Wang et al. [10], which made them differ from ageing research on the lab-aged materials. Rejuvenators were designed by Zhang et al. [11] and Su et al. [12] using petroleum technology and encapsulating rejuvenator fiber, and then added into recycled pavement material and lab-aged material. They concluded that rejuvenators can soften aged pavement materials and consequently recover the road performance. Furthermore, self-healing characteristics of pavement materials were reported by Wu et al. [13], Li et al. [14], and Cai et al. [15]. Wu et al. [13] found that UV irradiation will weaken the macro-structure and lower the failure strength and healing index. Li et al. [14] designed steel fiber modified asphalt concrete to promote the induction heating technology, while Cai et al. [15] used engineered cementitious composites mortar to prepare flexible pavement materials with certain healing property. Numerical simulation models of microwave heating of asphalt mixture, which can be used for pavement maintenance, recycling, and deicing, were developed with finite element software by Wang et al. [16].

Environmental conditions such as higher temperature, UV radiation, and moisture can introduce significant deteriorations of asphalt-based pavement materials. Materials modification technologies are thus widely used in pavement engineering to improve the long-term performance of pavement materials. For instance, ethylene bis stearamide based graphene [17], styrene-butadiene-styrene latex [18], styrene-butadiene rubber [19], aged lignin [20] and bio-based polyurethane [21] were used as modifier and detailed explained in this special issue. The viscos-elastic behavior, storage property, fracture energy, rutting resistance, and anti-cracking property were presented. Studies on high-viscosity modified asphalt binder [22] and fire-retardant asphalt [23] were also discussed in this special issue.

Characterization research on pavement materials is of important for this Special Issue. Performance studies on stress absorbing membrane interlayer and semi-flexible composite mixture were discussed by Yang et al. [24] and Zhang et al. [25]. The former article investigated the phase transition characteristics by dynamic mechanical analysis, while the second article presented the engineering properties by means of thermal cracking, fatigue, rutting resistance, and moisture resistance. Asphalt-based materials are composed of binder, filler, and aggregates, and the interaction between each different compound is the key to get a better understanding of pavement performance. The effect of aggregate meso-structure on the permanent deformation of asphalt mixture was discussed by Zhang et al. [26], with the three-dimensional discrete element model. Their model can capture the aggregate morphologies of angularity, orientation, and surface texture. Chen et al. [27] investigated the asphalt-filler/aggregate interaction on self-designed interface specimens with dynamic shear rheometer. They concluded that asphalt mortar could be the closest subscale in terms of performance to that of asphalt mixtures, making it a vital scale to bridge the gap between asphalt binder and asphalt mixtures in multiscale performance analysis. A unified strength model, which can be used to overcome the design deviation caused by the randomness of the laboratory strength test and improve the accuracy degree, was described by Xia et al. [28]. Different loading stresses were investigated to conclude the unified strength model, as well as to study the asphalt mixture moduli in the research presented by Fan et al. [29].

Field investigation is the principal requirement to ensure safe and well-accepted driving conditions in pavement maintenance. In the study by Pan et al. [30], piezo-ultrasonic wave technology was used for damage detection, including groove damage and cylinder cutting damage, in road engineering. Pan found that factors such as temperature, defect size, and ultrasonic velocity would affect the detection accuracy. Zhang et al. [31] reported the field investigation in full-depth asphalt pavement.

Other researches focused on the wet skid resistance [32] and gradation design [33] of pavement surfacings. Lyu et al. [34] introduced an outstanding durable road surface marking material, using persistent phosphors coated with silica-polymer hybrid shell. Cool coating materials for asphalt pavements were designed and discussed by Chen et al. [35]. These studies are critically important for pavement design and highway service. For instance, the cool coating can be widely used to solve the high-temperature-related defects in asphalt pavement.

Several other studies involved in this Special Issue look at cement concrete materials. Cement concrete and asphalt concrete are the two major materials in pavement engineering, so-called rigid pavement and flexible pavement. The micro-zone corrosion mechanism [36], cement mortar with super absorbent polymer [37], and exhaust-purifying cement [38] were discussed in detail. Polypropylene fiber was used in cement concrete and its performance was studied by Chen et al. [39] by dynamic compressive behavior analysis. Last, but not least, Yan et al. [40] presented their excellent work on anti-corrosion property of glass flake, which was designed for the reinforcement in chemically bonding phosphate ceramic coatings.
